# Locus coeruleus degeneration is associated with cortical tau deposition and cognitive decline in older adults at familial risk of Alzheimer’s disease

**DOI:** 10.64898/2025.12.02.691923

**Published:** 2025-12-05

**Authors:** Alfie Wearn, Kate M. Onuska, Hayley R.C. Shanks, Rahul Gaurav, Stéphane Lehéricy, Giulia Baracchini, Colleen Hughes, Jennifer Tremblay-Mercier, Sridar Narayanan, John Breitner, Judes Poirier, Taylor W. Schmitz, Gary R. Turner, R. Nathan Spreng, the PREVENT-AD Research Group

## Abstract

**INTRODUCTION:**

The locus coeruleus (LC) is among the first sites of tau pathology in Alzheimer’s disease (AD) and may seed neocortical tau.

**METHODS:**

We used longitudinal neuromelanin-sensitive MRI to assess LC integrity *in vivo* in a cohort of cognitively unimpaired older adults with familial risk of AD in relation to tau and amyloid PET and long-term cognitive trajectories.

**RESULTS:**

We showed that both LC integrity at baseline and its rate of degeneration over time independently predicted a neocortical pattern of tau deposition. In keeping with the known function of the LC, neuropsychological tests showed that LC integrity at baseline predicted changes in attention. Finally, we found that longitudinal LC degeneration correlated with memory decline in people with elevated neocortical amyloid burden.

**DISCUSSION:**

Our findings underscore the importance of LC in AD pathogenesis. Longitudinal measurement of LC degeneration may help distinguish trajectories of age-related cognitive decline and early AD.

## Full Text Availability

The license terms selected by the author(s) for this preprint version do not permit archiving in PMC. The full text is available from the preprint server.

